# Methyl 6-dimethyl­amino-4-hy­droxy-2-naphtho­ate

**DOI:** 10.1107/S1600536810053237

**Published:** 2010-12-24

**Authors:** Jun Ho Do, Kwang-Jin Hwang, Moon-Hwan Kim, Chong-Hyeak Kim

**Affiliations:** a Department of Bio & Chemical Engineering, Hongik University, Jochiwon, Chungnam 339-701, Republic of Korea; b Biomaterial Research Center, Korea Research Institute of Chemical Technology, PO Box 107, Yuseong, Daejeon 305-600, Republic of Korea; c Center for Chemical Analysis, Korea Research Institute of Chemical Technology, PO Box 107, Yuseong, Daejeon 305-600, Republic of Korea

## Abstract

In the title compound, C_14_H_15_NO_3_, the ester group is oriented so that the carbonyl group points in the opposite direction to the hy­droxy group. The mol­ecule as a whole is almost planar (the r.m.s. deviation of the non-H atoms is 0.0268 Å). In the crystal, mol­ecules are linked by inter­molecular O—H⋯O hydrogen bonds into infinite chains that propagate parallel to the *c* axis.

## Related literature

For the synthesis, properties and applications of organic photochromic and thermochromic dyes, see: Gabbutt *et al.* (2003 ▶, 2004 ▶); Kim *et al.* (2010 ▶); Kumar *et al.* (1995 ▶); Gemert & Selvig (2000 ▶); Nelson *et al.* (2002 ▶). For an additional review of such materials, see; Crano & Guglielmetti (1999 ▶).
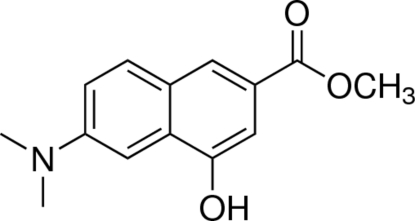


## Experimental

### 

#### Crystal data

C_14_H_15_NO_3_
*M* *_r_* = 245.27
Monoclinic, 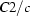

*a* = 27.2482 (5) Å
*b* = 6.6211 (1) Å
*c* = 13.6283 (3) Å
β = 97.203 (1)°
*V* = 2439.32 (8) Å^3^
*Z* = 8
Mo *K*α radiation
μ = 0.09 mm^−1^
*T* = 296 K
0.43 × 0.28 × 0.15 mm

#### Data collection

Bruker APEXII CCD diffractometer
11077 measured reflections
3019 independent reflections
2021 reflections with *I* > 2σ(*I*)
*R*_int_ = 0.023

#### Refinement

*R*[*F* ^2^ > 2σ(*F* ^2^)] = 0.047
*wR*(*F* ^2^) = 0.151
*S* = 1.05
3019 reflections
165 parameters
H-atom parameters constrained
Δρ_max_ = 0.23 e Å^−3^
Δρ_min_ = −0.18 e Å^−3^

### 

Data collection: *APEX2* (Bruker, 2009 ▶); cell refinement: *SAINT* (Bruker, 2009 ▶); data reduction: *SAINT*; program(s) used to solve structure: *SHELXS97* (Sheldrick, 2008 ▶); program(s) used to refine structure: *SHELXL97* (Sheldrick, 2008 ▶); molecular graphics: *SHELXTL* (Sheldrick, 2008 ▶); software used to prepare material for publication: *SHELXTL*.

## Supplementary Material

Crystal structure: contains datablocks global, I. DOI: 10.1107/S1600536810053237/pk2285sup1.cif

Structure factors: contains datablocks I. DOI: 10.1107/S1600536810053237/pk2285Isup2.hkl

Additional supplementary materials:  crystallographic information; 3D view; checkCIF report

## Figures and Tables

**Table 1:** Hydrogen-bond geometry (Å, °)

*D*—H⋯*A*	*D*—H	H⋯*A*	*D*⋯*A*	*D*—H⋯*A*
O15—H15*A*⋯O12^i^	0.82	1.92	2.736 (2)	170

Symmetry code: (i) 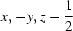.

**Fig. 1.**
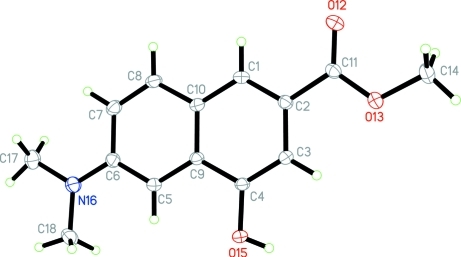
The molecular structure of the title compound with the atomic numbering scheme and 30% probability displacement ellipsoids. H atoms are shown as small spheres of arbitary radius.

**Fig. 2.**
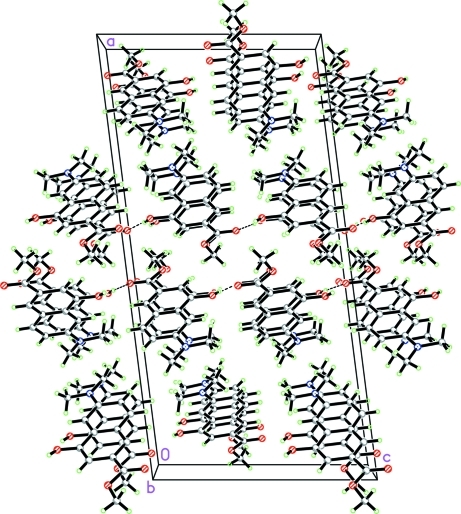
The molecular packing of the title compound, viewed down the b axis showing the O—H···O (dashed lines) hydrogen bonds.

## supplementary crystallographic information

### Comment

The synthesis and applications of organic photochromic and thermochromic dyes has become of great interest recently (Kumar *et al.*, 1995; Gemert & Selvig, 2000; Nelson *et al.*, 2002; Gabbutt *et al.*, 2003, 2004). These compounds may be useful as optical transmission materials in ophthalmic glasses and lenses. They have potential use in optical disks or memories (Crano & Guglielmetti, 1999). In the present work, the structure of methyl 6-(dimethylamino)-4-hydroxy-2-naphthoate has been determined to study the effect of substituents on the novel photochromic naphthopyrans (Kim *et al.*, 2010). The orientation of the hydroxy group and the carbonyl of the ester group in the structure of the title compound, C_14_H_15_NO_3_, are opposite to each other as shown in Fig. 1. The dimethylamino group, hydroxy group, methyl carboxyl group and the naphthonyl ring are almost coplanar (rms deviation = 0.0268 Å). In the crystal structure, the molecules are linked by moderate-strength intermolecular O—H···O hydrogen bonds into one-dimensional, infinite chains running along the *c* axis as shown in Fig. 2. The molecular chains are generated by O—H···O hydrogen bonds (Table 1) between the H atom of the hydroxy group and the O atom of the methyl carboxyl group.

### Experimental

Concentrated hydrochloric acid (3 ml) was added dropwise to a stirred solution of 4-acetoxy-6-dimethylamino-2-naphthonic acid (212.5 g) in methanol (1000 ml). On completion of the addition the solution was heated to reflux for 12 h and then cooled to room temperature. The resulting brown solution was evaporated and diluted with water (800 ml) and extracted with ethyl acetate (2 *x* 1200 ml). The organic extracts were dried over anhydrous magnesium sulfate and evaporated to give the title compound (164 g, yield 67%) as a white powder.  Single crystals suitable for X-ray diffraction were obtained from a solution in isopropyl alcohol.

### Refinement

All H atoms were placed in calculated positions using a riding model, with C—H = 0.93 Å and *U*_iso_(H) = 1.2 *U*_eq_(C) for aromatic H atoms, C—H = 0.96 Å and *U*_iso_(H) = 1.5 *U*_eq_(C) for methyl H atoms, and O—H = 0.82 Å and *U*_iso_(H) = 1.5 *U*_eq_(O) for hydroxy H atom.

### Crystal data

**Table:**

C_14_H_15_NO_3_	*F*(000) = 1040
*M**_r_* = 245.27	*D*_x_ = 1.336 Mg m^−^^3^
Monoclinic, *C*2/*c*	Mo *K*α radiation, λ = 0.71073 Å
Hall symbol: -C 2yc	Cell parameters from 3143 reflections
*a* = 27.2482 (5) Å	θ = 3.0–27.2°
*b* = 6.6211 (1) Å	µ = 0.09 mm^−^^1^
*c* = 13.6283 (3) Å	*T* = 296 K
β = 97.203 (1)°	Block, colorless
*V* = 2439.32 (8) Å^3^	0.43 × 0.28 × 0.15 mm
*Z* = 8	

### Data collection

**Table:**

Bruker APEXII CCD diffractometer	2021 reflections with *I* > 2σ(*I*)
Radiation source: fine-focus sealed tube	*R*_int_ = 0.023
graphite	θ_max_ = 28.3°, θ_min_ = 1.5°
φ and ω scans	*h* = −35→36
11077 measured reflections	*k* = −8→8
3019 independent reflections	*l* = −18→17

### Refinement

**Table:**

Refinement on *F*^2^	Primary atom site location: structure-invariant direct methods
Least-squares matrix: full	Secondary atom site location: difference Fourier map
*R*[*F*^2^ > 2σ(*F*^2^)] = 0.047	Hydrogen site location: inferred from neighbouring sites
*wR*(*F*^2^) = 0.151	H-atom parameters constrained
*S* = 1.05	*w* = 1/[σ^2^(*F*_o_^2^) + (0.0744*P*)^2^ + 0.749*P*] where *P* = (*F*_o_^2^ + 2*F*_c_^2^)/3
3019 reflections	(Δ/σ)_max_ < 0.001
165 parameters	Δρ_max_ = 0.23 e Å^−^^3^
0 restraints	Δρ_min_ = −0.18 e Å^−^^3^

### Special details

**Table:**

Geometry. All e.s.d.'s (except the e.s.d. in the dihedral angle between two l.s. planes) are estimated using the full covariance matrix. The cell e.s.d.'s are taken into account individually in the estimation of e.s.d.'s in distances, angles and torsion angles; correlations between e.s.d.'s in cell parameters are only used when they are defined by crystal symmetry. An approximate (isotropic) treatment of cell e.s.d.'s is used for estimating e.s.d.'s involving l.s. planes.
Refinement. Refinement of *F*^2^ against ALL reflections. The weighted *R*-factor *wR* and goodness of fit *S* are based on *F*^2^, conventional *R*-factors *R* are based on *F*, with *F* set to zero for negative *F*^2^. The threshold expression of *F*^2^ > σ(*F*^2^) is used only for calculating *R*-factors(gt) *etc*. and is not relevant to the choice of reflections for refinement. *R*-factors based on *F*^2^ are statistically about twice as large as those based on *F*, and *R*- factors based on ALL data will be even larger.

### Fractional atomic coordinates and isotropic or equivalent isotropic displacement parameters (Å^2^)

**Table:**

	*x*	*y*	*z*	*U*_iso_*/*U*_eq_	
C1	0.10637 (5)	0.2829 (2)	0.43577 (10)	0.0394 (4)	
H1A	0.1108	0.2627	0.5038	0.047*	
C2	0.07851 (5)	0.1492 (2)	0.37597 (10)	0.0362 (3)	
C3	0.07115 (5)	0.1797 (2)	0.27191 (10)	0.0373 (3)	
H3A	0.0521	0.0889	0.2314	0.045*	
C4	0.09206 (5)	0.3421 (2)	0.23145 (10)	0.0345 (3)	
C5	0.14347 (5)	0.6521 (2)	0.25090 (11)	0.0381 (3)	
H5A	0.1382	0.6726	0.1829	0.046*	
C6	0.17280 (5)	0.7870 (2)	0.30964 (11)	0.0401 (4)	
C7	0.18010 (6)	0.7510 (3)	0.41358 (12)	0.0468 (4)	
H7A	0.1999	0.8387	0.4545	0.056*	
C8	0.15860 (6)	0.5908 (3)	0.45420 (11)	0.0452 (4)	
H8A	0.1638	0.5726	0.5224	0.054*	
C9	0.12140 (5)	0.4845 (2)	0.29174 (10)	0.0335 (3)	
C10	0.12857 (5)	0.4515 (2)	0.39531 (10)	0.0363 (3)	
C11	0.05648 (5)	−0.0258 (2)	0.42183 (11)	0.0385 (3)	
O12	0.06137 (4)	−0.05793 (18)	0.51061 (8)	0.0511 (3)	
O13	0.03034 (4)	−0.14286 (17)	0.35582 (8)	0.0508 (3)	
C14	0.00549 (7)	−0.3150 (3)	0.39160 (15)	0.0580 (5)	
H14A	−0.0116	−0.3874	0.3366	0.087*	
H14B	−0.0178	−0.2700	0.4342	0.087*	
H14C	0.0294	−0.4024	0.4279	0.087*	
O15	0.08692 (4)	0.37976 (16)	0.13226 (7)	0.0451 (3)	
H15A	0.0791	0.2753	0.1019	0.068*	
N16	0.19479 (5)	0.9504 (2)	0.27050 (11)	0.0529 (4)	
C17	0.22463 (7)	1.0916 (3)	0.33220 (16)	0.0623 (5)	
H17A	0.2522	1.0219	0.3680	0.093*	
H17B	0.2050	1.1524	0.3781	0.093*	
H17C	0.2366	1.1946	0.2917	0.093*	
C18	0.18437 (7)	0.9976 (3)	0.16678 (14)	0.0583 (5)	
H18A	0.1965	0.8910	0.1286	0.087*	
H18B	0.2004	1.1220	0.1534	0.087*	
H18C	0.1493	1.0114	0.1492	0.087*	

### Atomic displacement parameters (Å^2^)

**Table:**

	*U*^11^	*U*^22^	*U*^33^	*U*^12^	*U*^13^	*U*^23^
C1	0.0450 (8)	0.0457 (9)	0.0270 (7)	0.0007 (6)	0.0030 (6)	0.0031 (6)
C2	0.0404 (7)	0.0346 (8)	0.0337 (7)	0.0021 (6)	0.0050 (6)	0.0038 (6)
C3	0.0454 (8)	0.0350 (7)	0.0310 (7)	−0.0005 (6)	0.0030 (6)	−0.0025 (6)
C4	0.0429 (7)	0.0352 (7)	0.0255 (7)	0.0038 (6)	0.0045 (6)	0.0001 (6)
C5	0.0448 (8)	0.0395 (8)	0.0304 (7)	0.0001 (6)	0.0059 (6)	0.0009 (6)
C6	0.0407 (7)	0.0397 (8)	0.0408 (8)	−0.0029 (6)	0.0085 (6)	−0.0004 (7)
C7	0.0495 (9)	0.0515 (10)	0.0388 (8)	−0.0119 (7)	0.0028 (7)	−0.0076 (8)
C8	0.0522 (9)	0.0539 (10)	0.0290 (7)	−0.0073 (7)	0.0030 (6)	−0.0025 (7)
C9	0.0377 (7)	0.0348 (7)	0.0286 (7)	0.0022 (6)	0.0062 (5)	−0.0011 (6)
C10	0.0404 (7)	0.0402 (8)	0.0285 (7)	0.0001 (6)	0.0045 (6)	−0.0019 (6)
C11	0.0418 (8)	0.0356 (8)	0.0383 (8)	0.0037 (6)	0.0063 (6)	0.0055 (6)
O12	0.0670 (7)	0.0479 (7)	0.0382 (6)	−0.0019 (6)	0.0065 (5)	0.0120 (5)
O13	0.0631 (7)	0.0452 (7)	0.0438 (6)	−0.0166 (5)	0.0054 (5)	0.0044 (5)
C14	0.0683 (11)	0.0434 (9)	0.0642 (12)	−0.0156 (8)	0.0160 (9)	0.0039 (9)
O15	0.0682 (7)	0.0412 (6)	0.0254 (5)	−0.0051 (5)	0.0039 (5)	−0.0003 (4)
N16	0.0618 (9)	0.0499 (8)	0.0470 (8)	−0.0190 (7)	0.0061 (7)	0.0030 (7)
C17	0.0618 (11)	0.0528 (11)	0.0721 (13)	−0.0179 (9)	0.0072 (9)	−0.0030 (10)
C18	0.0673 (11)	0.0511 (10)	0.0570 (11)	−0.0053 (9)	0.0092 (9)	0.0152 (9)

### Geometric parameters (Å, °)

**Table:**

C1—C2	1.367 (2)	C8—H8A	0.9300
C1—C10	1.414 (2)	C9—C10	1.417 (2)
C1—H1A	0.9300	C11—O12	1.219 (2)
C2—C3	1.422 (2)	C11—O13	1.325 (2)
C2—C11	1.479 (2)	O13—C14	1.442 (2)
C3—C4	1.365 (2)	C14—H14A	0.9600
C3—H3A	0.9300	C14—H14B	0.9600
C4—O15	1.364 (2)	C14—H14C	0.9600
C4—C9	1.428 (2)	O15—H15A	0.8200
C5—C6	1.385 (2)	N16—C17	1.439 (2)
C5—C9	1.410 (2)	N16—C18	1.441 (2)
C5—H5A	0.9300	C17—H17A	0.9600
C6—N16	1.376 (2)	C17—H17B	0.9600
C6—C7	1.426 (2)	C17—H17C	0.9600
C7—C8	1.362 (2)	C18—H18A	0.9600
C7—H7A	0.9300	C18—H18B	0.9600
C8—C10	1.414 (2)	C18—H18C	0.9600
			
C2—C1—C10	120.8 (1)	C8—C10—C9	117.6 (1)
C2—C1—H1A	119.6	C1—C10—C9	119.8 (1)
C10—C1—H1A	119.6	O12—C11—O13	123.7 (1)
C1—C2—C3	120.1 (1)	O12—C11—C2	123.8 (1)
C1—C2—C11	118.7 (1)	O13—C11—C2	112.5 (1)
C3—C2—C11	121.2 (1)	C11—O13—C14	117.9 (1)
C4—C3—C2	120.1 (1)	O13—C14—H14A	109.5
C4—C3—H3A	120.0	O13—C14—H14B	109.5
C2—C3—H3A	120.0	H14A—C14—H14B	109.5
O15—C4—C3	123.2 (1)	O13—C14—H14C	109.5
O15—C4—C9	115.5 (1)	H14A—C14—H14C	109.5
C3—C4—C9	121.3 (1)	H14B—C14—H14C	109.5
C6—C5—C9	121.6 (1)	C4—O15—H15A	109.5
C6—C5—H5A	119.2	C6—N16—C17	121.7 (1)
C9—C5—H5A	119.2	C6—N16—C18	120.6 (1)
N16—C6—C5	122.1 (1)	C17—N16—C18	117.4 (2)
N16—C6—C7	120.2 (1)	N16—C17—H17A	109.5
C5—C6—C7	117.7 (1)	N16—C17—H17B	109.5
C8—C7—C6	121.4 (1)	H17A—C17—H17B	109.5
C8—C7—H7A	119.3	N16—C17—H17C	109.5
C6—C7—H7A	119.3	H17A—C17—H17C	109.5
C7—C8—C10	121.7 (1)	H17B—C17—H17C	109.5
C7—C8—H8A	119.2	N16—C18—H18A	109.5
C10—C8—H8A	119.2	N16—C18—H18B	109.5
C5—C9—C10	120.1 (1)	H18A—C18—H18B	109.5
C5—C9—C4	121.9 (1)	N16—C18—H18C	109.5
C10—C9—C4	118.0 (1)	H18A—C18—H18C	109.5
C8—C10—C1	122.6 (1)	H18B—C18—H18C	109.5
			
C10—C1—C2—C3	−0.6 (2)	C7—C8—C10—C9	−0.1 (2)
C10—C1—C2—C11	179.4 (1)	C2—C1—C10—C8	−178.5 (1)
C1—C2—C3—C4	0.3 (2)	C2—C1—C10—C9	0.9 (2)
C11—C2—C3—C4	−179.8 (1)	C5—C9—C10—C8	−0.5 (2)
C2—C3—C4—O15	179.4 (1)	C4—C9—C10—C8	178.6 (1)
C2—C3—C4—C9	−0.2 (2)	C5—C9—C10—C1	−179.9 (1)
C9—C5—C6—N16	179.6 (1)	C4—C9—C10—C1	−0.8 (2)
C9—C5—C6—C7	0.0 (2)	C1—C2—C11—O12	0.5 (2)
N16—C6—C7—C8	179.8 (2)	C3—C2—C11—O12	−179.5 (1)
C5—C6—C7—C8	−0.7 (2)	C1—C2—C11—O13	179.9 (1)
C6—C7—C8—C10	0.7 (3)	C3—C2—C11—O13	−0.1 (2)
C6—C5—C9—C10	0.6 (2)	O12—C11—O13—C14	1.5 (2)
C6—C5—C9—C4	−178.5 (1)	C2—C11—O13—C14	−177.9 (1)
O15—C4—C9—C5	−0.1 (2)	C5—C6—N16—C17	179.1 (2)
C3—C4—C9—C5	179.6 (1)	C7—C6—N16—C17	−1.4 (2)
O15—C4—C9—C10	−179.2 (1)	C5—C6—N16—C18	5.7 (2)
C3—C4—C9—C10	0.5 (2)	C7—C6—N16—C18	−174.8 (2)
C7—C8—C10—C1	179.2 (2)		

### Hydrogen-bond geometry (Å, °)

**Table:**

*D*—H···*A*	*D*—H	H···*A*	*D*···*A*	*D*—H···*A*
O15—H15A···O12^i^	0.82	1.92	2.736 (2)	170

Symmetry codes: (i) *x*, −*y*, *z*−1/2.
